# Insulin resistance and chronic kidney disease progression, cardiovascular events, and death: findings from the chronic renal insufficiency cohort study

**DOI:** 10.1186/s12882-019-1220-6

**Published:** 2019-02-20

**Authors:** Sarah J. Schrauben, Christopher Jepson, Jesse Y. Hsu, F. Perry Wilson, Xiaoming Zhang, James P. Lash, Bruce M. Robinson, Raymond R. Townsend, Jing Chen, Leon Fogelfeld, Patricia Kao, J. Richard Landis, Daniel J. Rader, L. Lee Hamm, Amanda H. Anderson, Harold I. Feldman

**Affiliations:** 1 Division of Renal-Electrolyte and Hypertension, Department of Medicine, Perelman School of Medicine at the University of Pennsylvania, Philadelphia, 19103 PA USA; 2 Center for Clinical Epidemiology and Biostatistics, Perelman School of Medicine at the University of Pennsylvania, Philadelphia, PA 19103 USA; 3 Department of Biostatistics, Epidemiology, and Informatics, Perelman School of Medicine at the University of Pennsylvania, Philadelphia, PA USA; 4 Department of Medicine, Yale School of Medicine, New Haven, CT USA; 5 Department of Medicine, University of Illinois at Chicago College of Medicine, Chicago, IL USA; 6 Department of Internal Medicine, University of Michigan Medical School, Ann Arbor, MI; Arbor Research Collaborative for Health, Ann Arbor, MI USA; 7 Department of Medicine, Tulane University School of Medicine, New Orleans, Lousiana USA; 8 Department of Medicine, Rush University Medical Center, Chicago, IL USA; 9 Deparment of Medicine, Washington University School of Medicine in Saint Louis, St. Louis, MO USA; 10 Tulane University, School of Public Health and Tropical Medicine, New Orleans, LA USA

**Keywords:** Insulin resistance, Chronic kidney disease, Chronic renal insufficiency, Cardiovascular disease, Mortality

## Abstract

**Background:**

Insulin resistance contributes to the metabolic syndrome, which is associated with the development of kidney disease. However, it is unclear if insulin resistance independently contributes to an increased risk of chronic kidney disease (CKD) progression or CKD complications. Additionally, predisposing factors responsible for insulin resistance in the absence of diabetes in CKD are not well described. This study aimed to describe factors associated with insulin resistance and characterize the relationship of insulin resistance to CKD progression, cardiovascular events and death among a cohort of non-diabetics with CKD.

**Methods:**

Data was utilized from Chronic Renal Insufficiency Cohort Study participants without diabetes (*N* = 1883). Linear regression was used to assess associations with insulin resistance, defined using the Homeostasis Model Assessment of Insulin Resistance (HOMA-IR). The relationship of HOMA-IR, fasting glucose, hemoglobin A1c (HbA1c), and C-peptide with CKD progression, cardiovascular events, and all-cause mortality was examined with Cox proportional hazards models.

**Results:**

Novel positive associations with HOMA-IR included serum albumin, uric acid, and hemoglobin A1c. After adjustment, HOMA-IR was not associated with CKD progression, cardiovascular events, or all-cause mortality. There was a notable positive association of one standard deviation increase in HbA1c with the cardiovascular endpoint (HR 1.16, 95% CI: 1.00–1.34).

**Conclusion:**

We describe potential determinants of HOMA-IR among a cohort of non-diabetics with mild-moderate CKD. HOMA-IR was not associated with renal or cardiovascular events, or all-cause mortality, which adds to the growing literature describing an inconsistent relationship of insulin resistance with CKD-related outcomes.

**Electronic supplementary material:**

The online version of this article (10.1186/s12882-019-1220-6) contains supplementary material, which is available to authorized users.

## Background

Chronic kidney disease (CKD) affects up to 26 million Americans, resulting in a disproportionate risk of cardiovascular disease and end-stage renal disease (ESRD) [1]. The strong association of CKD with cardiovascular disease is explained only in part by traditional risk factors, and it is hypothesized that metabolic or inflammatory abnormalities associated with progressive renal disease, such as insulin resistance, confer an augmented risk of cardiovascular disease in CKD [2–5]. Insulin resistance is a pathological state in which tissues have a decreased sensitivity to insulin, leading to a compensatory rise in circulating insulin to maintain normal blood glucose levels [6–9]. Increased levels of insulin have been reported to be an important risk factor for the development of atherosclerosis in the general population [10, 11].

Insulin resistance is present in the early stages of CKD [12–15], and becomes more prevalent as CKD progresses [16]. However, the precise mechanisms of insulin resistance in CKD remains poorly identified [8]. Further, due to the exclusion of individuals with CKD in many epidemiologic studies of insulin resistance, it is unclear if insulin resistance alone contributes to an increased risk of important clinical outcomes in CKD [17].

In this study, we examined factors associated with insulin resistance in CKD and investigated the association of insulin resistance, and markers of carbohydrate metabolism, with subsequent CKD progression, atherosclerotic cardiovascular events, and all-cause mortality among individuals with mild-moderate CKD without diabetes.

## Methods

### Study design and population

The Chronic Renal Insufficiency Cohort (CRIC) Study is a prospective observational cohort study that enrolled a total of 3939 men and women with CKD across the United States between 2003 and 2008 at seven clinical centers (Ann Arbor, Michigan; Baltimore, Maryland; Chicago, Illinois; Cleveland, Ohio; New Orleans, Louisiana; Philadelphia, Pennsylvania; and Oakland, California) with age-specific estimated glomerular filtration rate (eGFR) criteria ranging 20–70 mL/min/1.73m^2^. Eligibility criteria have been previously reported [18, 19]. Participants completed annual clinic visits at which data were obtained, and blood and urine specimens were collected. Diabetes mellitus was defined as a fasting glucose > 7 mmol/L, a non-fasting glucose > 11.1 mmol/L, or the use of insulin or other medications for glycemic control. Study participants without diabetes at baseline and with a fasting blood draw were included (*N* = 1883). The study protocol was approved by the Institutional Review Board of all participating centers and is in accordance with the Declaration of Helsinki. All participants provided written informed consent.

#### Exposures

The primary exposure was insulin resistance, using the Homeostasis Model Assessment of Insulin Resistance (HOMA-IR), estimated from fasting glucose and insulin values from the baseline visit [20]. HOMA-IR is the most commonly used static test of insulin sensitivity. Additional measures were evaluated as secondary exposures, including fasting glucose, hemoglobin A1c (HbA1c), and C-peptide.

#### Outcomes and censoring events

The composite renal endpoint was defined as the development of ESRD (dialysis initiation or kidney transplantation) or halving of baseline eGFR. Estimated GFR was calculated from serum creatinine and cystatin C using the CRIC Study equation [21]. The composite atherosclerotic cardiovascular endpoint included the first hospitalization for a myocardial infarction, a cerebrovascular event, or peripheral arterial disease. Hospitalizations were ascertained through self-report and adjudicated by study personnel. Deaths were ascertained from reports of next of kin, death certificates, obituaries, hospital records, and the Social Security Death Master File. Follow-up was censored at time of death or loss to follow-up. Outcomes were ascertained through 2015.

#### Covariates

All considered covariates were ascertained at the baseline visit, which included age, sex, race, ethnicity, level of education, systolic blood pressure (SBP), smoking status (current vs. other), physical activity (minutes/week), waist circumference (in centimeters), body mass index (BMI, kg/m^2^), fat-free mass (FFM), self-reported history of cardiovascular disease, statin use, other non-statin lipid-lowering medication use, angiotensin-converting enzyme inhibitor (ACEi) or angiotensin-receptor blocker (ARB) use, eGFR, hemoglobin, high-density lipoprotein (HDL) cholesterol, low-density lipoprotein (LDL) cholesterol, triglycerides, high-sensitivity C-reactive protein (hsCRP), uric acid, serum albumin, fibroblast growth factor 23 (FGF-23), and 24-h proteinuria. Further details of the data collection procedures are provided in the supplement.

### Statistical analysis

Baseline characteristics of study participants were summarized overall and across quartiles of HOMA-IR using frequencies for categorical variables, and either mean (standard deviation [SD]) or median (interquartile range [IQR]) for continuous variables, as appropriate. Data were transformed if the distribution was skewed. Differences in characteristics across HOMA-IR quartiles were compared using analysis of variance, chi-square test, and Kruskal Wallis test, as appropriate. Correlations between the main predictor (HOMA-IR) and measures of carbohydrate metabolism (C-peptide, HbA1c, and glucose) were explored with Pearson’s correlation coefficient, *r*. The association of HOMA-IR (using the transformed version, log_2_-HOMA-IR) with factors reported to be associated with insulin resistance in non-diabetics with CKD, which include age, smoking status, SBP, BMI, HDL, LDL, triglycerides, ACEi/ARB use, physical activity, hemoglobin, eGFR, hsCRP, waist circumference, and FFM was assessed with multivariable adjusted linear regression models controlled for race/ethnicity, sex, clinical center, and education. An exploratory linear regression further adjusted the model for: history of cardiovascular disease, use of statins, use of non-statin lipid-lowering medications, 24-h proteinuria, HbA1c, uric acid, FGF-23, and serum albumin. Participants with no missing data were included in the primary and exploratory linear regression analyses (*N* = 1806 and *N* = 1706, respectively).

Median follow-up time, total number of events, and crude rates of the renal endpoint, cardiovascular endpoint, and all-cause death were calculated overall and by quartiles of HOMA-IR. The association of HOMA-IR, fasting glucose, HbA1c, and C-peptide with each of the endpoints was examined using traditional Cox proportional hazards models with sequential adjustment. Exposures of interest were modeled per 1 SD increase. Model 1 adjusted for age, sex, race, ethnicity, education, and clinical center. Model 2 adjusted for Model 1 covariates, plus BMI, waist circumference, smoking status, SBP, ACEi/ARB use, HDL, LDL, triglycerides, hsCRP, FFM, eGFR, hemoglobin, and physical activity. Finally, Model 3 adjusted for Model 2 covariates with the addition of statin use, use of non-statin lipid-lowering medications, history of cardiovascular disease, 24-h proteinuria, FGF-23, uric acid, and serum albumin. Hazard ratios (HR) and 95% confidence intervals (95% CI) were reported for all models. Analyses were restricted to cases with full data (*N* = 1882 in Model 1, *N* = 1806 in Model 2, and *N* = 1706 in Model 3).

We explored effect modification by an a priori selected set of characteristics: race (Black vs. non-Black), age (< 65 vs. ≥65 years), eGFR (< 45 vs. ≥45 mL/min/1.73m^2^), and proteinuria (< 0.2 vs. ≥0.2 g/day). In sensitivity analyses, Cox proportional hazards models of the cardiovascular endpoint were repeated treating death as a competing event, and the models of the cardiovascular endpoint and all-cause mortality were repeated censoring at ESRD. All analyses were performed using SAS® software, version 9.4 (SAS Institute Inc., Cary, NC) [22].

## Results

A total of 1883 CRIC Study participants did not have diabetes at study entry. These participants had a mean age of 56.5 years, a mean BMI of 30.3 kg/m^2^, and a mean eGFR of 49 mL/min/1.73m^2^. Mean values for HOMA-IR, glucose, insulin, HbA1c, and C-peptide were 3.84, 5 mmol/L, 0.6 μg/L, 39 mmol/mol, and 3.0 μg/L, respectively. Baseline characteristics are reported by quartile of HOMA-IR in Table 1. (Due to the positively skewed nature of the distribution of HOMA-IR values, which causes Quartile 4 to contain a much wider range of values than Quartiles 1–3, that quartile is divided in two.) History of cardiovascular disease, BMI, waist circumference, statin use, hsCRP, glucose, insulin, HbA1c, and C-peptide all increased with the upper quartiles of HOMA-IR. Notably, components of the metabolic syndrome (central obesity as measured by waist circumference, fasting glucose, triglycerides, and HDL) trended as expected with increasing quartiles of HOMA-IR. HOMA-IR was strongly correlated with C-peptide (*r* = 0.78), moderately correlated with glucose (*r* = 0.54), and weakly correlated with HbA1c (*r* = 0.26).

**Table 1:** Baseline characteristics of Chronic Renal Insufficiency Cohort (CRIC) Study participants without diabetes at baseline overall and by quartile of HOMA-IR

		Quartile of HOMA-IR, mmol/L * μU/mL					
	Overall	Quartile 1	Quartile 2	Quartile 3	Quartile 4a	Quartile 4b	*P*-value
		(0.04–2.04)	(2.05–3.02)	(3.03–4.55)	(4.56–6.20)	(6.21–41.1)	
	*N* = 1883	*N* = 471	*N* = 471	*N* = 471	*N* = 235	*N* = 235	
Demographic/Clinical variables							
Age, years	56.5 (11.9)	54.2 (12.9)	56.9 (11.6)	57.7 (11.4)	57.2 (11.6)	57.2 (10.7)	< 0.001
Male gender, %	1011 (54.0)	232 (49.3)	241 (51.2)	273 (58.0)	136 (57.9)	129 (54.9)	0.04
Race/ethnicity, %							0.03
Non-Hispanic white	939 (50.0)	258 (54.8)	231 (49.0)	244 (51.8)	104 (44.3)	102 (43.4)	
Non-Hispanic black	724 (38.0)	155 (32.9)	174 (36.9)	181 (38.4)	103 (43.8)	111 (47.2)	
Hispanic	144 (8.0)	38 (8.1)	42 (8.9)	31 (6.6)	17 (7.2)	16 (6.8)	
Hypertension, %	1503 (80.0)	321 (68.2)	370 (78.6)	397 (84.3)	205 (87.2)	210 (89.4)	< 0.001
Current smoking, %	258 (14.0)	82 (17.4)	65 (13.8)	45 (9.6)	36 (15.3)	30 (12.8)	0.01
Systolic blood pressure, mmHg	124 (20)	122 (21)	124 (21)	125 (20)	123 (19)	124 (18)	0.16
Diastolic blood pressure, mmHg	73 (13)	73 (13)	73 (13)	73 (12)	73 (13)	73 (13)	0.93
Heart rate, beats/min	67 (11)	65 (11)	66 (11)	67 (11)	67 (10)	69 (11)	< 0.001
History of cardiovascular disease, %	452 (24)	81 (17)	110 (23)	126 (27)	64 (27)	71 (30)	< 0.001
Education, %							
Less than high school	268 (14)	57 (12.1)	72 (15.3)	74 (15.7)	28 (11.9)	37 (15.7)	< 0.001
High school graduate	343 (18)	69 (14.7)	86 (18.3)	84 (18.5)	52 (22.1)	49 (20.9)	
Some college	541 (29)	115 (24.5)	138 (29.3)	126 (26.8)	73 (31.1)	89 (37.9)	
College graduate or higher	730 (39)	229 (48.7)	175 (37.2)	184 (39.1)	82 (34.9)	60 (25.5)	
Anthropometric Variables							
Height, m	1.7 (0.1)	1.7 (0.1)	1.7 (0.1)	1.7 (0.1)	1.7 (0.1)	1.7 (0.1)	0.006
Weight, kg	87 (21.7)	74.9 (16.5)	81.7 (17.4)	91.8 (20.3)	96.9 (21.2)	102.1 (25)	< 0.001
Body mass index, kg/m^2^	30.3 (7.1)	26.4 (4.9)	28.8 (6.0)	31.7 (6.2)	33.4 (6.8)	35.8 (8.7)	< 0.001
Waist circumference, cm	101.6 (16.3)	91.8 (13.8)	97.6 (13.0)	105.1 (13.7)	109.9 (15.2)	114.3 (17.6)	< 0.001
Fat-free mass, kg	57.4 (14.5)	52.2 (12.6)	54.8 (12.8)	60.2 (15.2)	61.7 (14.5)	63.0 (15.2)	< 0.001
Medication Use							
Statins, %	778 (42)	132 (28.1)	201 (43.3)	223 (47.8)	108 (46)	114 (48.5)	< 0.001
Other lipid-lowering medication, %	855 (46)	146 (31.1)	219 (47.2)	244 (52.2)	121 (51.5)	125 (53.2)	< 0.001
ACEi or ARB use, %	1092 (58%)	219 (46.7)	273 (58.8)	287 (61.5)	163 (69.4)	150 (63.8)	
Laboratory Values							
Fasting glucose, mmol/L	5 (0.6)	4.7 (0.6)	4.9 (0.5)	5.2 (0.6)	5.4 (0.6)	5.7 (0.6)	< 0.001
Insulin, μg/L	0.6 (0.4–0.9)	0.4 (0.3–0.4)	0.5 (0.5–0.6)	0.8 (0.7–0.8)	1.0 (0.9–1.1)	1.6 (1.3–2.0)	< 0.001
C-peptide, μg /L	3.0 (2.1–4.1)	1.8 (1.4–2.3)	2.5 (2.0–3.1)	3.4 (2.9–4.1)	4.1 (3.5–4.0)	5.8 (5.7–7.3)	< 0.001
eGFR, mL/min/1.73m^2^	49 (18)	51 (20)	49 (18)	48 (16)	48 (18)	46 (15)	0.010
24-h urine protein, g/day							
< 0.10	838 (46)	227 (50)	203 (45.1)	201 (43.7)	99 (44.6)	108 (48.4)	< 0.05
0.10–0.49	543 (30)	131 (29)	147 (32.7)	153 (33.3)	59 (26.6)	53 (23.8)	
0.50–1.49	242 (13)	55 (12)	66 (14.7)	54 (11.7)	31 (14)	36 (16.1)	
≥1.50	188 (10)	43 (9)	34 (7.6)	52 (11.3)	33 (14.9)	26 (11.7)	
Serum albumin, g/L	41 (4)	40 (5)	41 (4)	41 (4)	40 (4)	41 (4)	0.001
Hemoglobin, mmol/L	8.2 (1.1)	8.0 (1.1)	8.1 (1.1)	8.2 (1.1)	8.2 (1.1)	8.3 (1.1)	0.004
Total cholesterol, mmol/L	4.9 (1.1)	5.0 (1.1)	4.9 (1.1)	4.8 (1.0)	4.9 (1.0)	4.9 (1.2)	< 0.05
LDL, mmol/L	2.8 (0.9)	2.9 (0.9)	2.8 (0.9)	2.8 (0.8)	2.8 (0.8)	2.7 (0.9)	0.02
HDL, mmol/L	1.3 (0.4)	1.4 (0.5)	1.4 (0.4)	1.2 (0.4)	1.1 (0.3)	1.1 (0.3)	< 0.001
Triglycerides, mmol/L	1.4 (1.0–1.9)	1.1 (0.8–1.6)	1.3 (0.9–1.7)	1.4 (1.1–2.0)	1.6 (1.2–2.4)	1.7 (1.2–2.8)	< 0.001
Hemoglobin A1c, mmol/mol	39	37	38	39	41	41	< 0.001
Uric acid, umol/L	428.3 (113.0)	398.5 (113.0)	416.4 (113.0)	434.2 (107.1)	458 (107.1)	475.8 (107.1)	
High-sensitivity CRP, nmol/L	22.9 (9.5–58.1)	14.3 (7.6–34.3)	21.0 (7.6–53.3)	26.7 (11.4–62.9)	32.4 (14.3–75.2)	39.0 (14.3–81.0)	< 0.001
FGF-23, relative units (RU)/mL	121.8 (84.0–199.2)	111.9 (76.2–189.9)	117.0 (80.6–181.2)	128.3 (87.3–206.6)	130.4 (91.3–222.0)	136.0 (91.0–234.4)	< 0.001

Values in the table are expressed as mean (SD), median (25th–75th percentile), or N (%)

*HOMA-IR* – homeostasis model assessment insulin resistance; ACEi- angiotensin-converting enzyme inhibitor, *ARB* – angiotensin receptor blocker, *eGFR* – estimated glomerular filtration rate, *LDL* – low density lipoprotein, *HDL* – high density lipoprotein, *CRP* – C-reactive protein, *FGF-23* – fibroblast growth factor 23

Several baseline characteristics were significantly associated with log_2_-HOMA-IR (Tables 2 and 3): age per year (β = 0.005, *p* = 0.001), non-smoking vs. current smoking (β = 0.13, *p* = 0.002), BMI per kg/m^2^ (β = 0.02, *p* < 0.001), waist circumference per centimeter (β = 0.01, *p* < 0.001), hemoglobin per mmol/L (β = 0.04, *p* < 0.001), LDL per mmol/L (β = − 0.002 *p* < 0.001), HDL per mmol/L (β = − 0.003, *p* = 0.001), triglycerides per mmol/L (β = 0.30, *p* < 0.001), and hsCRP per nmol/L (β = 0.04, *p* = 0.03). In the exploratory linear regression, the following were found to be associated with HOMA-IR: use of non-statin lipid lowering medications vs. non-use (β = − 0.16, *p* = 0.03), serum albumin per g/L (β = 0.12, *p* = 0.001), HbA1c per mmol/mol (β = 0.2, *p* < 0.001), and uric acid per umol/L (β =0.03, *p* = 0.001).

**Table 2:** Multivariable-adjusted association of HOMA-IR and factors previously reported to be associated with insulin resistance -- Chronic Renal Insufficiency Cohort (CRIC) Study participants without diabetes at baseline (*N* = 1806)

Demographic/Clinical variables	β	*P*
Age, years	0.005	0.001
Non-smoking	0.13	0.002
Systolic blood pressure, mmHg	−0.001	0.28
Physical activity^a^	− 0.0001	0.43
Anthropometric Variables		
Body mass index, kg/m^2^	0.02	< 0.001
Waist circumference, cm	0.01	< 0.001
Fat-free mass, kg	−0.003	0.15
Medication Use		
No ACEi or ARB use	−0.05	0.11
Laboratory Values		
eGFR, mL/min/1.73m^2^	−0.00001	0.99
Hemoglobin, mmol/L	0.04	< 0.001
LDL, mmol/L	−0.002	< 0.001
HDL, mmol/L	−0.003	0.001
(log) Triglycerides, mmol/L	0.30	< 0.001
(log) High-sensitivity CRP, nmol/L	0.04	0.03

^a^Physical activity measured as total reported METS in one week

Model also adjusted for sex, race, ethnicity, and education

*HOMA-IR* – homeostasis model assessment insulin resistance, *ACEi*- angiotensin-converting enzyme inhibitor, *ARB* – angiotensin receptor blocker, *eGFR* – estimated glomerular filtration rate, *LDL* – low density lipoprotein, *HDL* – high density lipoprotein, *CRP* – C-reactive protein

**Table 3:** Exploratory multivariable-adjusted association of HOMA-IR and novel factors -- Chronic Renal Insufficiency Cohort (CRIC) Study participants without diabetes at baseline (*N* = 1706)

Clinical variables	β	*P*
No history of cardiovascular disease	0.01	0.86
Medication Use		
No statin use	0.11	0.14
Non-statin lipid-lowering medication use	−0.16	0.03
Laboratory Values		
Proteinuria, g/day		
< 0.10	−0.07	0.20
0.2 to < 0.5	−0.07	0.20
0.5 to < 1.5	−0.002	0.97
Hemoglobin A1c, per %	0.20	< 0.001
Uric acid, umol/L	0.03	< 0.001
(log) FGF-23, relative units (RU)/mL	0.04	0.10
Serum albumin, g/L	0.12	0.001

Model adjusted for age, sex, race, ethnicity, and education, smoking status, systolic blood pressure, physical activity, fat free mass, body mass index, waist circumference, angiotensin-converting enzyme inhibitor or angiotensin receptor blocker use;, hemoglobin level, estimated glomerular filtration rate– low density lipoprotein, high density lipoprotein; triglycerides C-reactive protein

The median (IQR) duration of follow-up was 7.76 (3.40–10.26) years for the composite renal endpoint, with 474 renal events (event rate 3.68 per 100 person-years), 9.43 (5.95–10.81) years for the composite cardiovascular endpoint with 220 cardiovascular events (event rate: 1.43 per 100 person-years), and 10.15 (9.74–11.09) years for mortality with 379 deceased during follow-up (event rate: 2.19 per 100 person-years; Table 4). The assumption of proportionality was met in the fully adjusted Cox models.

**Table 4:** Mean follow-up time, total numbers of events, and crude event rates of renal and atherosclerotic cardiovascular composite endpoints and all-cause mortality by quartile of HOMA-IR among CRIC participants without diabetes at baseline

		Quartile of HOMA-IR				
	Overall	Quartile 1	Quartile 2	Quartile 3	Quartile 4a	Quartile 4b
Event: Composite Renal Endpoint (ESRD/Halving of eGFR)						
Sample size	1883	471	471	471	235	235
# of events	474	114	109	126	64	61
Median (IQR) follow up, years	7.76 (3.40–10.26)	7.65 (3.06–10.30)	7.88 (3.24–10.35)	7.76 (3.93–10.18)	7.30 (3.57–10.03)	7.84 (3.32–10.16)
Event rate per 100 person-years (95% CI)	3.68 (3.37–4.03)	3.55 (2.96–4.27)	3.39 (2.81–4.09)	3.85 (3.23–4.58)	4.08 (3.20–4.58)	3.80 (2.96–4.88)
Event: Composite Atherosclerotic CVD Endpoint (MI/PAD/stroke)						
Sample size	1883	471	471	471	235	235
# of events	220	47	53	55	33	32
Median (IQR) follow up, years	9.43 (5.95–10.81)	9.41 (6.14–10.90)	9.51 (5.86–10.70)	9.45 (7.03–10.87)	9.51 (6.10–10.70)	9.23 (5.43–10.59)
Event rate per 100 person-years (95% CI)	1.43 (1.25–1.63)	1.22 (0.92–1.62)	1.38 (1.05–1.80)	1.39 (1.07–1.82)	1.73 (1.23–2.43)	1.72 (1.22–2.44)
Event: All-Cause Mortality						
Sample size	1883	471	471	471	235	235
# of events	379	96	90	98	44	51
Median (IQR) follow up, years	10.15 (8.74–11.09)	10.18 (8.66–11.10)	10.00 (8.66–11.02)	10.25 (8.83–11.14)	10.21 (8.98–11.05)	9.74 (8.60–11.01)
Event rate per 100 person-years (95% CI)	2.19 (1.98–2.42)	2.21 (1.81–2.71)	2.11 (1.72–2.60)	2.23 (1.83–2.72)	2.00 (1.49–2.69)	2.40 (1.83–3.16)

Abbreviations: *CVD* – cardiovascular disease, *eGFR* – estimated glomerular filtration rate, *ESRD* – end-stage renal disease, *HOMA-IR* – homeostasis model assessment insulin resistance, *IQR* – interquartile range, *MI* – myocardial infarction, *PAD* – peripheral arterial disease, *CI*- confidence interval

HOMA-IR was not significantly associated with CKD progression, atherosclerotic cardiovascular events, or all-cause mortality (Table 5). For each 1 SD higher in HbA1c there was a 16% greater rate of the cardiovascular endpoint (HR 1.16, 95% CI: 1.00–1.34). The finding was consistent in adjusted models censored at ESRD (HR 1.25, 95% CI 1.06–1.47; Additional file 1 Table S1) and when modeling death as a competing event (HR 1.18, 95% CI: 1.01–1.39; Additional file 1 Table S2). For each 1 SD higher fasting glucose, there was a 12% lower rate of CKD progression (HR 0.88, 95% CI: 0.79–0.98).

**Table 5:** Multivariable-adjusted hazard ratios of renal and atherosclerotic cardiovascular composite endpoints and all-cause mortality per 1 SD increase in HOMA-IR and other markers of carbohydrate metabolism among CRIC participants without diabetes at baseline

	Model 1^b^			Model 2^b^			Model 3^b^	
	HR^a^	95% CI		HR^a^	95% CI		HR*	95% CI
Event: Composite Renal Endpoint (ESRD/Halving of eGFR)								
HOMA-IR	0.97	0.89–1.07		1.01	0.90–1.12		1.01	0.90–1.14
Glucose	0.93	0.85–1.03		0.94	0.85–1.04		0.88	0.79–0.98
HbA1c	0.98	0.89–1.07		1.00	0.90–1.10		0.94	0.85–1.04
C-peptide	1.32	1.20–1.44		1.16	1.03–1.31		1.02	0.90–1.17
Event: Composite Atherosclerotic CVD Endpoint (MI/PAD/stroke)								
HOMA-IR	1.04	0.91–1.19		1.04	0.89–1.22		1.00	0.85–1.19
Glucose	1.04	0.91–1.20		1.06	0.91–1.23		1.05	0.90–1.23
HbA1c	1.22	1.07–1.39		1.22	1.06–1.40		1.16	1.00–1.34
C-peptide	1.17	1.02–1.34		1.07	0.89–1.29		1.02	0.84–1.24
Event: All-Cause Mortality								
HOMA-IR	0.94	0.85–1.05		1.01	0.89–1.14		0.98	0.86–1.12
Glucose	0.96	0.87–1.07		0.99	0.89–1.11		0.99	0.88–1.11
HbA1c	1.03	0.93–1.14		1.05	0.94–1.17		0.99	0.88–1.10
C-peptide	1.18	1.06–1.31		1.16	1.01–1.33		1.09	0.94–1.27

Abbreviations: *CI* – confidence interval, *CVD* – cardiovascular disease, *eGFR* – estimated glomerular filtration rate, *ESRD* – end-stage renal disease, *HbA1c* – glycosylated hemoglobin, *HOMA-IR* – homeostasis model assessment insulin resistance, *HR* – hazard ratio, *MI* – myocardial infarction, *PAD* – peripheral arterial disease

^a^Per 1 standard deviation increase

Model 1 includes adjustment for age, sex, race, ethnicity, level of education, and clinical center

Model 2 includes adjustment for variables in Model 1 plus body mass index, waist circumference, smoking status, systolic BP, ACEi/ARB use, HDL, LDL, triglycerides, high sensitivity CRP, fat-free mass, eGFR, hemoglobin, physical activity

Model 3 includes adjustment for variables in Model 2 plus use of statins, use of other lipid-lowering medications, history of CVD, 24-h urine protein, FGF-23, uric acid, serum albumin

^b^Sample sizes: Model 1 (*N* = 1882), Model 2 (*N* = 1806), Model 3 (*N* = 1706)

For CKD progression and all-cause death, there were no significant interactions of the prespecified variables (race, age, proteinuria) and HOMA-IR. For the composite cardiovascular endpoint, age (< 65 yrs. vs. ≥65 years) significantly modified the effect of HOMA-IR (*p* = 0.0003). The age-stratified results demonstrated a trend toward a greater hazard for the cardiovascular endpoint with greater HOMA-IR among those < 65 years (HR 1.19, 95% CI: 0.98–1.45) and a lower hazard for those ≥65 years (HR 0.50, 95% CI: 0.35–0.72).

## Discussion

In the present study, we examined factors that potentially contribute to insulin resistance, as measured by HOMA-IR, in individuals with mild-to-moderate CKD in the absence of diabetes, and investigated the association of HOMA-IR and other carbohydrate metabolism measures with CKD progression, atherosclerotic cardiovascular events, and all-cause mortality. We observed that age, current smoking, greater BMI and waist circumference, and higher hemoglobin, triglycerides and hsCRP were independently associated with HOMA-IR, which is consistent with prior reports. HOMA-IR was not significantly associated with CKD progression, atherosclerotic cardiovascular events, or all-cause mortality in adjusted models.

The current study is among the first to report significant associations of uric acid, serum albumin, HbA1c, and the use of non-statin lipid-lowering medications with HOMA-IR among non-diabetics with CKD. These associations were independent of age, sex, race, education and other risk factors, such as SBP, BMI, physical activity and eGFR. The use of non-statin lipid-lowering medications was negatively associated with HOMA-IR, a finding consistent with the prior observation that higher cholesterol levels have been associated with HOMA-IR [23, 24]. Hyperuricemia has been demonstrated to be strongly associated with abnormal glucose metabolism and insulin resistance, but to our knowledge this association has not been reported in nondiabetics with CKD [25, 26]. The positive association of HbA1c and HOMA-IR suggests chronic mild hyperglycemia and decreased sensitivity to insulin is present even in the absence of overt diabetes in those with mild-moderate CKD. Higher serum albumin levels and insulin resistance have also been associated in nondiabetic populations without kidney disease [27, 28]. Higher serum albumin in the setting of insulin resistance is thought to be the consequence of increased albumin production caused by insulin stimulation [29].

Many of the factors found to be independently associated with HOMA-IR in CKD are consistent with previous reports, in particular with body composition measures, which include BMI and fat mass in CKD patients without diabetes [30–32, 12, 24, 30, 33]. It is postulated that the higher levels of inflammatory mediators found in visceral fat contributes to the development of insulin resistance [34, 35], which is consistent with our finding that waist circumference, BMI, and hsCRP were greater within higher quartiles of HOMA-IR. Systemic inflammation is thought to contribute to decreased tissue sensitivity to insulin and the increased risk of cardiovascular disease in CKD by driving endothelial dysfunction and atherosclerosis [36].

Despite the proposed link between insulin resistance, endothelial dysfunction, and atherosclerosis, the association of insulin resistance and cardiovascular events has not been consistent in the setting of kidney disease [12, 30, 37–39]. A study by Shinohara et al. demonstrated that HOMA-IR was an independent predictor of cardiovascular mortality in nondiabetics with ESRD [37], but insulin resistance among those with earlier CKD, assessed with the gold standard for insulin resistance, the hyperinsulinemic euglycemic glucose clamp (HEGC) technique [30], or HOMA-IR [40], was not associated with new cardiovascular events. In the present study, we did not find HOMA-IR to be associated with the composite cardiovascular disease outcome. Interestingly, we found a trend for higher levels of HbA1c to be associated with an increased hazard of the cardiovascular endpoint, which suggests that mild elevations in blood glucose over time, even in the absence of overt diabetes, increase the risk of cardiovascular events in the setting of mild-moderate CKD. This finding is striking given that the positive association was observed in a range where we would not expect higher risk, and a recent report from the CRIC study found that HbA1c levels were not associated with incident type 2 diabetes [41]. A potential explanation could be that we included individuals with pre-diabetes into our study sample since enrollment blood glucose levels were below the level of exclusion. In a recent meta-analysis, pre-diabetes in the general population was associated with an increased risk of cardiovascular disease [42]. Mild hyperglycemia has also been reported to contribute to atherosclerosis in apparently healthy subjects and was independently associated with greater arterial stiffness in the CRIC Study [43, 44].

Hyperinsulinemia has been reported to influence kidney function by inducing glomerular hyperfiltration, endothelial dysfunction, and increased vascular permeability [45, 46]. However, there have been contradictory reports on the association of insulin resistance and CKD progression in those without diabetes. In several studies of populations with CKD, insulin resistance was associated with a more rapid decline in kidney function compared to those who were insulin sensitive [47, 40, 48]. However, in a prospective study of 73 non-diabetic participants with CKD, there was not a significant difference in eGFR between those who did and did not have insulin resistance (as measured by HOMA-IR) [49]. In the current study, we did not find an association of HOMA-IR, HbA1c, or C-peptide with CKD progression. Interestingly, higher levels of glucose were associated with decreased CKD progression, which is unexpected since HbA1c, the marker of prolonged hyperglycemia, was not associated with CKD progression. This finding should be further explored with longitudinal measures to better define the relationship.

Prior studies have not clearly demonstrated that insulin resistance predicts all-cause mortality in CKD [30, 37, 39]. In a cohort of 170 ESRD patients in Japan without diabetes, HOMA-IR predicted mortality independently of other risk factors, including inflammation and BMI [37], but in a cohort of nearly all non-diabetic elderly Caucasian men with mild-to-moderate CKD, insulin resistance was not associated with all-cause death, independent of classical risk factors. In the current study, HOMA-IR and the other markers of carbohydrate metabolism were not significantly associated with all-cause mortality.

The overall lack of association between HOMA-IR and important clinical endpoints in this study could be due to an imperfect estimate of insulin resistance in CKD. We utilized measures that largely reflect hepatic insulin resistance (i.e., fasting insulin and glucose) to calculate HOMA-IR, which does not capture peripheral insulin resistance (i.e. skeletal muscle) which is thought to be the primary location for insulin resistance in CKD [50]. A validation study of insulin sensitivity surrogates using a study sample of 1074 men, 495 with CKD (median eGFR 46 ml/min per 1.73m^2^) and without CKD deemed HOMA-IR a satisfactory surrogate as compared to HEGC technique [38]. However, the findings of the validation study might not extend to our study population since it was conducted in eldery men (age 70–71 years) from one region of Sweden with less obesity (all with BMI < 30 kg/m^2^).

The observational nature of the CRIC Study and the cross sectional analysis in identifying potential determinants of HOMA-IR are limitations of the current study, and do not allow us to establish causality. Since HOMA-IR was assessed only at baseline, we are not able to investigate for changes in HOMA-IR over the follow up period and subsequently, the impact of a change in HOMA-IR would have on the clinical outcomes of interest. Further, misclassification of diabetes status is also possible as it did not incorporate a measure of HbA1c.

Strengths of this study include the wealth of data collected through validated measures, which allowed for the assessment of associations of insulin resistance and other markers of carbohydrate metabolism that have been minimally explored, as well as the description of novel factors associated with insulin resistance in a population with mild-to-moderate CKD without diabetes. Additional strengths include the evaluation of the health implications of HOMA-IR over a broad range of eGFR values and across multiple racial/ethnic groups, which expands the generalization from other studies that were carried out on highly selected populations (e.g., only male or only Caucasian) with limited range of eGFR.

## Conclusions

The results of the current study of participants with mild-moderate CKD in the absence of diabetes have demonstrated novel factors positively associated with HOMA-IR, including serum albumin, HbA1c, and uric acid. We also found consistent associations of body composition measures and systemic inflammation with HOMA-IR. HOMA-IR, fasting glucose, and C-peptide were not significantly associated with development of renal or atherosclerotic cardiovascular events, or all-cause mortality, which adds to the growing literature describing an inconsistent relationship of insulin resistance with important CKD-related outcomes. Assessment of peripheral rather than hepatic insulin resistance may better define the role of insulin resistance in mild-moderate CKD. Increased HbA1c was associated with an elevated hazard of the cardiovascular endpoint, suggesting that mild hyperglycemia, even in the absence of overt diabetes, may increase the risk of cardiovascular events in CKD. This finding should be further explored with longitudinal measures to better define the relationship.

## Additional file

Additional file 1: **Table S1.** Multivariable-adjusted hazard ratios of atherosclerotic cardiovascular composite endpoints and all-cause mortality per 1 SD increase in HOMA-IR and other markers of carbohydrate metabolism among CRIC participants without diabetes at baseline, censored at ESRD for CVD and death outcomes. **Table S2.** Multivariable-adjusted hazard ratios of atherosclerotic cardiovascular composite endpoints per 1 SD increase in HOMA-IR and other markers of carbohydrate metabolism among CRIC participants without diabetes at baseline modeling death as a competing event. (PDF 91 kb)

## Abbreviations

ACEi: angiotensin-converting enzyme inhibitor
ARB: angiotensin-receptor blocker
BMI: body mass index
CKD: Chronic kidney disease
CRIC: Chronic Renal Insufficiency Cohort
ESRD: End-stage renal disease
FFM: fat-free mass
FGF-23: fibroblast growth factor-23
HbA1c: Hemoglobin A1c
HDL: high-density lipoprotein
HOMA-IR: HOMA-IR- Homeostasis Model Assessment of Insulin Resistance
hsCRP: high sensitivity C-reactive protein
IQR: interquartile range
LDL: low-density lipoprotein
SBP: Systolic blood pressure
SD: standard deviation
Study; eGFR: estimated glomerular filtration rate
