# Supplementary material for: Insulin resistance and chronic kidney disease progression, cardiovascular events, and death: findings from the chronic renal insufficiency cohort study
Source: BMC Nephrol. 2019 Feb 20;20:60. doi: 10.1186/s12882-019-1220-6 (PMC6383235; doi:10.1186/s12882-019-1220-6)
Supplement: Supplementary file 1 — Table S1. Multivariable-adjusted hazard ratios of atherosclerotic cardiovascular composite endpoints and all-cause mortality per 1 SD increase in HOMA-IR and other markers of carbohydrate metabolism among CRIC participants without diabetes at baseline, censored at ESRD for CVD and death outcomes. Table S2. Multivariable-adjusted hazard ratios of atherosclerotic cardiovascular composite endpoints per 1 SD increase in HOMA-IR and other markers of carbohydrate metabolism among CRIC participants without diabetes at baseline modeling death as a competing event. (PDF 91 kb) [file 12882_2019_1220_MOESM1_ESM.pdf]

Supplemental Material

Data Collection Procedures

Systolic blood pressure was measured with the participant seated using a Tycos™ Classic hand aneroid cuff and sphygmomanometer. Glucose was detected using a coupled enzymatic method (www.orthoclinical.com). Insulin and C-peptide were measured in EDTA plasma using radioimmunoassay (www.millipore.com). Hemoglobin A1c was analyzed in whole blood using a boronate affinity high-performance liquid chromatography (HPLC) method (www.trinitybiotech.com) for samples collected through February 2006, and an ion exchange HPLC method (www.bio-rad.com) for samples collected subsequently. A complete blood count was analyzed at each of the CRIC clinical sites using local clinical laboratories.

Serum creatinine was measured at the CRIC Central Laboratory by an enzymatic method (www.orthoclinical.com) for samples collected through October 2008 and by the Jaffe method (www.beckmancoulter.com) thereafter, and standardized to isotope dilution mass spectrometry-traceable values.[24;25] Serum cystatin C was measured using a particle-enhanced immunonephelometric assay on the Siemens BN™ II System (www.siemens.com).

Table S1. Multivariable-adjusted hazard ratios of atherosclerotic cardiovascular composite endpoints and all-cause mortality per 1 SD increase in HOMA-IR and other markers of carbohydrate metabolism among CRIC participants without diabetes at baseline, censored at ESRD for CVD and death outcomes

	Model 1		Model 2		Model 3	
	**HR***	**95% CI**	**HR***	**95% CI**	**HR***	**95% CI**
Event: Composite Atherosclerotic CVD Endpoint (MI/PAD/stroke)						
HOMA-IR	1.01	0.87-1.18	1.01	0.84-1.21	0.98	0.81-1.20
Glucose	1.08	0.93-1.25	1.12	0.95-1.31	1.13	0.96-1.35
HbA1c	1.31	1.14-1.51	1.31	1.34-1.53	1.25	1.06-1.47
C-peptide	1.16	0.99-1.35	1.08	0.88-1.32	1.06	0.85-1.31
Event: All-Cause Mortality						
HOMA-IR	0.91	0.80-1.04	0.95	0.82-1.10	0.92	0.78-1.07
Glucose	0.96	0.84-1.09	1.00	0.87-1.14	1.01	0.88-1.17
HbA1c	1.02	0.90-1.16	1.07	0.93-1.22	1.01	0.88-1.17
C-peptide	1.10	0.96-1.26	1.10	0.92-1.31	1.07	0.89-1.29

Abbreviations: CI – confidence interval; CRP – C-reactive protein; CVD – cardiovascular disease; ESRD – end-stage renal disease; HbA1c – glycosylated hemoglobin; HDL – high density lipoprotein; HOMA-IR – homeostasis model assessment insulin resistance; HR – hazard ratio; MI – myocardial infarction; PAD – peripheral arterial disease

* Per 1 standard deviation increase

Model 1 includes adjustment for age, sex, race, ethnicity, level of education, and clinical center

Model 2 includes adjustment for variables in Model 1 plus body mass index, waist circumference, smoking status, systolic BP, ACEi/ARB use, HDL, LDL, triglycerides, high sensitivity CRP, fat-free mass, eGFR, hemoglobin, physical activity

Model 3 includes adjustment for variables in Model 2 plus use of statins, use of other lipid-lowering medications, history of CVD, 24-hr urine protein, FGF-23, uric acid, serum albumin

Table S2. Multivariable-adjusted hazard ratios of atherosclerotic cardiovascular composite endpoints per 1 SD increase in HOMA-IR and other markers of carbohydrate metabolism among CRIC participants without diabetes at baseline modeling death as a competing event.

	**Model 1**		**Model 2**		**Model 3**	
	**HR***	**95% CI**	**HR***	**95% CI**	**HR***	**95% CI**
Event: Composite Atherosclerotic CVD Endpoint (MI/PAD/stroke)						
HOMA-IR	1.04	0.91-1.20	1.04	0.89-1.23	1.02	0.85-1.21
Glucose	1.05	0.92-1.20	1.07	0.91-1.25	1.07	0.91-1.26
HbA1c	1.23	1.07-1.41	1.23	1.06-1.43	1.18	1.01-1.39
C-peptide	1.14	1.00-1.30	1.08	0.91-1.29	1.01	0.85-1.26

Abbreviations: CI – confidence interval; CRP – C-reactive protein; CVD – cardiovascular disease; HbA1c – glycosylated hemoglobin; HOMA-IR – homeostasis model assessment insulin resistance; HR – hazard ratio; MI – myocardial infarction; PAD – peripheral arterial disease
* Per 1 standard deviation increase
Model 1 includes adjustment for age, sex, race, ethnicity, level of education, and clinical center
Model 2 includes adjustment for variables in Model 1 plus body mass index, waist circumference, smoking status, systolic BP, ACEi/ARB use, HDL, LDL, triglycerides, high sensitivity CRP, fat-free mass, eGFR, hemoglobin, physical activity
Model 3 includes adjustment for variables in Model 2 plus use of statins, use of other lipid-lowering medications, history of CVD, 24-hr urine protein, FGF-23, uric acid, serum albumin
